# Effectiveness and cost-effectiveness of home palliative care services for adults with advanced illness and their caregivers

**DOI:** 10.1590/1516-3180.20161341T2

**Published:** 2016-01-19

**Authors:** Barbara Gomes, Natalia Calanzani, Vito Curiale, Paul McCrone P, Irene J. Higginson, Maja de Brito

## Abstract

**BACKGROUND:**

Extensive evidence shows that well over 50% of people prefer to be cared for and to die at home provided circumstances allow choice. Despite best efforts and policies, one-third or less of all deaths take place at home in many countries of the world.

**OBJECTIVES:**

1. to quantify the effect of home palliative care services for adult patients with advanced illness and their family caregivers on patients’ odds of dying at home; 2. to examine the clinical effectiveness of home palliative care services on other outcomes for patients and their caregivers such as symptom control, quality of life, caregiver distress and satisfaction with care; 3. to compare the resource use and costs associated with these services; 4. to critically appraise and summarize the current evidence on cost-effectiveness.

**METHODS:**

*Search methods:* We searched 12 electronic databases up to November 2012. We checked the reference lists of all included studies, 49 relevant systematic reviews, four key textbooks and recent conference abstracts. We contacted 17 experts and researchers for unpublished data.

*Selection criteria:* We included randomised controlled trials (RCTs), controlled clinical trials (CCTs), controlled before and after studies (CBAs) and interrupted time series (ITSs) evaluating the impact of home palliative care services on outcomes for adults with advanced illness or their family caregivers, or both.

*Data collection and analysis:* One review author assessed the identified titles and abstracts. Two independent reviewers performed assessment of all potentially relevant studies, data extraction and assessment of methodological quality. We carried out meta-analysis where appropriate and calculated numbers needed to treat to benefit (NNTBs) for the primary outcome (death at home).

**MAIN RESULTS:**

We identified 23 studies (16 RCTs, 6 of high quality), including 37,561 participants and 4042 family caregivers, largely with advanced cancer but also congestive heart failure (CHF), chronic obstructive pulmonary disease (COPD), HIV/AIDS and multiple sclerosis (MS), among other conditions. Meta-analysis showed increased odds of dying at home (odds ratio (OR) 2.21, 95% CI 1.31 to 3.71; Z = 2.98, P value = 0.003; Chi2 = 20.57, degrees of freedom (df) = 6, P value = 0.002; I2 = 71%; NNTB 5, 95% CI 3 to 14 (seven trials with 1222 participants, three of high quality)). In addition, narrative synthesis showed evidence of small but statistically significant beneficial effects of home palliative care services compared to usual care on reducing symptom burden for patients (three trials, two of high quality, and one CBA with 2107 participants) and of no effect on caregiver grief (three RCTs, two of high quality, and one CBA with 2113 caregivers). Evidence on cost-effectiveness (six studies) is inconclusive.

**AUTHORS’ CONCLUSIONS:**

The results provide clear and reliable evidence that home palliative care increases the chance of dying at home and reduces symptom burden in particular for patients with cancer, without impacting on caregiver grief. This justifies providing home palliative care for patients who wish to die at home. More work is needed to study cost-effectiveness especially for people with non-malignant conditions, assessing place of death and appropriate outcomes that are sensitive to change and valid in these populations, and to compare different models of home palliative care, in powered studies.

The full text of this review is available free of charge from: http://onlinelibrary.wiley.com/doi/10.1002/14651858.CD007760.pub2/abstract
